# 961. Prevalence of Surgical Site Infections and Bacteremia in Pediatric Patients Receiving Extracorporeal Membrane Oxygentaion

**DOI:** 10.1093/ofid/ofad500.022

**Published:** 2023-11-27

**Authors:** Samiyah Siraj Althagafi, Mohammed Abdullah Alsuhaibani, Mashael Murdhi Alenzi, Ohoud Abdulrahaman Alyabes, Reem Mohammed Beheri, Fahad Khalid Alrawaf, Afnan Abdullah Almalki, Raghad Tariq Alhuthil

**Affiliations:** Taif’s Children Hospital, Riyadh, Ar Riyad, Saudi Arabia; King Faisal Specialist Hospital & Research Centre, Riyadh, Ar Riyad, Saudi Arabia; King Salman Armed Forces Hospital, Riyadh, Ar Riyad, Saudi Arabia; King Faisal Specialist Hospital & Research Centre, Riyadh, Ar Riyad, Saudi Arabia; King Faisal Specialist Hospital & Research Centre, Riyadh, Ar Riyad, Saudi Arabia; King Faisal Specialist Hospital & Research Centre, Riyadh, Ar Riyad, Saudi Arabia; King Faisal Specialist Hospital & Research Centre, Riyadh, Ar Riyad, Saudi Arabia; King Faisal Specialist Hospital & Research Centre, Riyadh, Ar Riyad, Saudi Arabia

## Abstract

**Background:**

Extracorporeal membrane oxygenation (ECMO) is a well-known type of advanced life support used in critically ill patients with serious heart and/or lung problems. ECMO treatment is associated with multiple life-threatening complications. The objectives of this study were to identify the prevalence of surgical site infections (SSI) and bloodstream infections (BSI) along with the associated risk factors and outcomes in patients on ECMO.

**Methods:**

In this retrospective study, clinical charts of patients were reviewed from January 2012 to April 2023. All admitted pediatric patients 14 years or less who received ECMO treatment were enrolled. Mann-Whitney and Fisher exact tests were used to assess mortality-related risk factors.

**Results:**

A total of 165 patients were included in the study, 79 (47.88%) females and 86 (52.12%) males, with a median age of 4 [IQR 0.8-24] months. BSI were found in 33 (20%) of them, 10 (30.30%) were gram-positive, 25 (75.75%) were gram-negative, and 6 (18.18%) were fungi. Furthermore, 33 (20%) developed SSI. The overall mortality rate in this study is (50.30%) with a median duration from ECMO procedure initiation to death being 11 [IQR: 5-26] days. Most of mortalities (78.31%) were noticed in infants aged ≤24 months. Compared with patient with neither SSI nor BSI, patients with SSI and BSI had a higher mortality rates of 19 (22.89%) and 16 (19.28%) respectively.

Additionally, other noted complications were mediastinitis 4 (2.42%), sternal osteomyelitis 2 (1.21%), infective endocarditis 4 (2.42%).

Younger age and thrombocytopenia were found to be a mortality related risk factors (P value 0.04 and 0.01 respectively).

**Figure:**
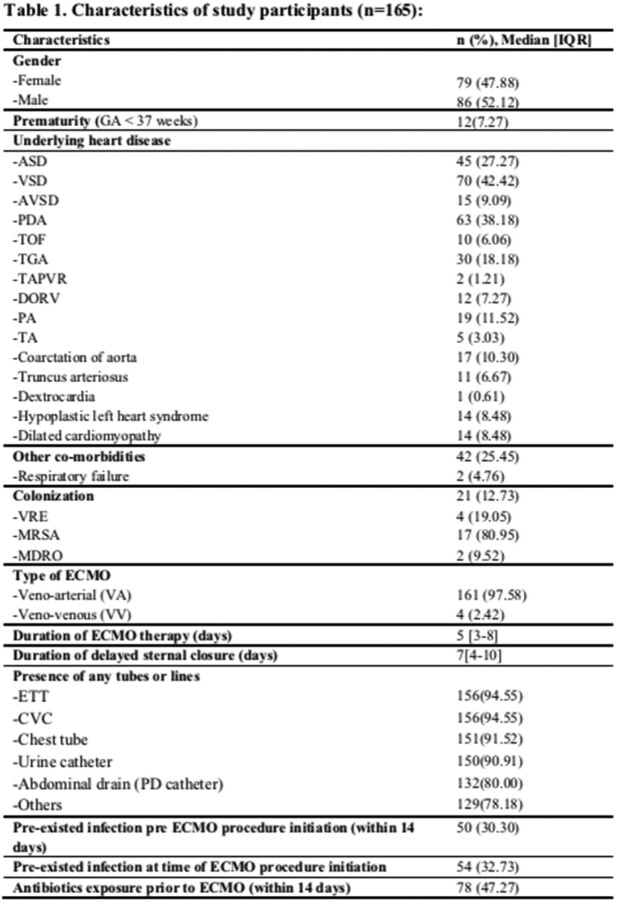

**Conclusion:**

Our study found a high rate of bacteremia and SSI in pediatric patients receiving ECMO. Younger age, thrombocytopenia were the potential risk factors associated with higher mortality rates in this group.

**Disclosures:**

**All Authors**: No reported disclosures

